# Pharmacokinetics of Azalomycin F, a Natural Macrolide Produced by Streptomycete Strains, in Rats

**DOI:** 10.3390/molecules26216464

**Published:** 2021-10-26

**Authors:** Su He, Wenjia Zhao, Peibo Li, Wenqing Tu, Kui Hong, Duoduo Zhang, Tongke Zhang, Ganjun Yuan

**Affiliations:** 1 Biotechnological Engineering Center for Pharmaceutical Research and Development, Jiangxi Agricultural University, Nanchang 330045, China; suhepeilin@gmail.com (S.H.); 18770910458@sohu.com (W.Z.); blossom_zhang@sohu.com (D.Z.); ztk2628925331@sohu.com (T.Z.); 2 Guangdong Engineering and Technology Research Center for Quality and Efficacy Re-Evaluation of Post-Marketed TCM, State Key Laboratory of Biocontrol and Guangdong Provincial Key Laboratory of Plant Resources, School of Life Sciences, Sun Yat-sen University, Guangzhou 510275, China; lipeibo@mail.sysu.edu.cn; 3 Research Institute of Poyang Lake, Jiangxi Academy of Sciences, Nanchang 330012, China; tuwenqing@jxas.ac.cn; 4 Key Laboratory of Combinatorial Biosynthesis and Drug Discovery, Ministry of Education, School of Pharmaceutical Sciences, Wuhan University, Wuhan 430071, China; kuihong31@whu.edu.cn

**Keywords:** azalomycin F, pharmacokinetics, rat, bioavailability, macrolide, antimicrobial agent

## Abstract

As antimicrobial resistance has been increasing, new antimicrobial agents are desperately needed. Azalomycin F, a natural polyhydroxy macrolide, presents remarkable antimicrobial activities. To investigate its pharmacokinetic characteristics in rats, the concentrations of azalomycin F contained in biological samples, in vitro, were determined using a validated high-performance liquid chromatography–ultraviolet (HPLC-UV) method, and, in vivo, samples were assayed by an ultra-high performance liquid chromatography–tandem mass spectrometric (UPLC–MS/MS) method. Based on these methods, the pharmacokinetics of azalomycin F were first investigated. Its plasma concentration-time courses and pharmacokinetic parameters in rats were obtained by a non-compartment model for oral (26.4 mg/kg) and intravenous (2.2 mg/kg) administrations. The results indicate that the oral absolute bioavailability of azalomycin F is very low (2.39 ± 1.28%). From combinational analyses of these pharmacokinetic parameters, and of the results of the in-vitro absorption and metabolism experiments, we conclude that azalomycin F is absorbed relatively slowly and with difficulty by the intestinal tract, and subsequently can be rapidly distributed into the tissues and/or intracellular f of rats. Azalomycin F is stable in plasma, whole blood, and the liver, and presents plasma protein binding ratios of more than 90%. Moreover, one of the major elimination routes of azalomycin F is its excretion through bile and feces. Together, the above indicate that azalomycin F is suitable for administration by intravenous injection when used for systemic diseases, while, by oral administration, it can be used in the treatment of diseases of the gastrointestinal tract.

## 1. Introduction

Antimicrobial resistance has emerged as a serious threat to human health and economic development, and new antimicrobial agents are desperately need [1,2]. Azalomycin F, a 36-membered polyhydroxy macrolide produced by many streptomycete strains [3,4,5,6], has various bioactivities against Gram-positive bacteria, yeast, fungi, and protozoa [3,6,7,8,9], and even some clinical studies on its anti-trichomoniasis and anti-candida infectious effects have been performed [3,10]. Moreover, it presents remarkably inhibitory activity of the interleukin-1 receptor [11,12,13]. This complex contains three main components, the azalomycins F_3a_, F_4a_ and F_5a_ [4,5,6,7], whose plane structures were established in 1959 and revised in 2011 [4]. Then, their relative configurations were first established in 2013 [14], and a review on these polyhydroxy macrolides was presented by us in 2019 [3]. Based on chemical and genomic analyses, the absolute configurations of their analogs niphimycins were proposed in 2018 [15], and their absolute configurations were subsequently suggested in 2021 [16].

The antimicrobial mechanisms indicated that azalomycin F can increase the cell-membrane permeability of *Staphylococcus aureus* through the synergistic effects of its lactone ring binding to the polar head of the cell-membrane phospholipid and its guanidyl side chain targeting to lipoteichoic acid (LTA), eventually leading to the autolysis of *S. aureus* cells [17,18]. As LTA plays an essential role in bacterial growth, cell division, biofilm formation, autolysin regulation, and resistance to cationic antibiotics [19,20], it has already been considered as an important target for new antimicrobial agents [20,21,22]. So, that azalomycin F targets Gram-positive bacterial cell-membrane phospholipids and LTAs indicates that it has a great potential to be developed into a new antimicrobial agent. 

Compositional analysis has indicated that phosphatidylglycerol (PG), lysyl-phosphatidylglycerol (LPG) and cardiolipin are the three major components of the *S. aureus* cell-membrane phospholipid [23,24], and that the content of LPG increases when *S. aureus* becomes resistant to daptomycin [23]. As many publications have reported [25,26], molecular dynamics (MD) simulation has been widely used for revealing the detailed interactions between drug molecules and membrane lipids. Two systems, respectively containing 1,2-dihexadecanoyl-*sn*-glycero-3-phospho-(1′-*rac*-glycerol) (DPPG)/azalomycin F_5a_ and DPPG/lysyl-DPPG/azalomycin F_5a,_ were used for MD simulation in our previous work [18], and the results showed that azalomycin F_5a_ had a greater adhesive force to plasma membranes assembled by DPPG plus lysyl-DPPG than by DPPG. This suggested that azalomycin F_5a_ likely has a greater antagonistic activity to daptomycin-resistant *S. aureus* than to daptomycin-susceptible one. 

Recently, it was also discovered that azalomycin F can eradicate the mature biofilm of *S. aureus* [27]. As the nature of this biofilm can remarkably increase the antimicrobial resistance, as compared to their planktonic counterparts and probably leads to the bacterial resistance to antimicrobials [28,29,30,31,32], these, together, suggest that azalomycin F may offer many advantages in the research and development of drugs against resistant bacteria. Recently, our review on the chemistry, biology, and structure–activity relationship of these guanidine-containing polyhydroxyl macrolides showed that many bioactivities were continuously reported for azalomycin F and its analogs, and also indicated that azalomycin F can be considered a lead compound or a potential drug to be further explored [3]. Here, the pharmacokinetics of azalomycin F are investigated for the first time for its druggability, and for ascertaining possible drug-delivery routes.

## 2. Materials and Methods

### 2.1. Chemicals and Reagents

Azalomycin F (purity, 98%) was isolated from the fermentation of *Streptomyces hygroscopicus* var. *azalomyceticus* according to our published methods [4]. Acetonitrile and methyl alcohol of HPLC grade were obtained from Fisher Scientific (Pittsburgh, PA, USA), formic acid was purchased from Sigma–Aldrich (St. Louis, MA, USA). Deionized water was purified using a TST-UP water purification system (HBTest, Hebei, China). All the other reagents were of analytical grade. Before use, azalomycin F (59.4 mg) was dissolved in 900 μL DMSO. To the solution, 17.1 mL of 0.5% sodium carboxymethyl cellulose (CMC-Na) was dropwise added and immediately mixed to obtain the suspension for intragastric administration at a dose of 26.4 mg/kg. In another solution, azalomycin F (6.5 mg) was completely dissolved in 400 μL DMSO, to which 7.6 mL of 2% Tween 80 in normal saline solution was slowly added, and immediately mixed into the solution for intravenous administration through the tail vein of rats at a dose of 2.2 mg/kg.

### 2.2. Animal

Male Sprague–Dawley rats (weighing 200 ± 20 g) were provided by the Experimental Animal Center of Jiangxi University of Traditional Chinese Medicine (Jiangxi, China). These rats were housed in controlled conditions (temperature of 22 ± 2 °C, humidity of (50 ± 10)%, 12 h light/dark cycle), and fed standard rat chow and drinking water, freely, for a week prior to the experiments. All animal experiments were carried out according to the Guidelines for Animal Experimentation of Jiangxi Agricultural University; the procedure was approved by the Animal Ethics Committee of Jiangxi Agricultural University with the project identification code JXAULL-2019010 on 5 March 2019. For vena caudal administration, 75% alcohol was used to first wipe one of the caudal veins at distance of about 1/3 to 1/2 of the whole tail length from its end. After the vein had swelled, approximate 1.0 mL of fresh azalomycin F solution was injected at a dose of 2.2 mg/kg, and the injection site was immediately pressed with a cotton ball to stop bleeding. Before retroorbital sampling, diethyl ether was used anesthetize the rats by inhalation. During the anesthesia process, the breathing state and complexion of rats were closely attended for the timely adjusting their lying posture, to prevent excessive anesthesia or asphyxial death. At designated time points, 200 μL of blood was collected from each rat.

### 2.3. LC-MS/MS Analysis and Method Validation

The quantification method for azalomycin F in rat plasma was developed using ultra-performance liquid chromatography tandem mass spectrometric technology (UPLC-MS/MS). Briefly, azalomycin F was analyzed by an Agilent 1290 Infinity HPLC System consisting of an Agilent 6420 Triple Quadrupole (Agilent, CA, USA), and an Eclipase Plus C_18_ column (3.0 mm × 50 mm, 1.8 μm, Agilent) was used. Eighty percent acetonitrile (water/acetonitrile, 1:4 (*v*/*v*)), containing 0.1% formic acid, was used as the mobile phase. The column temperature was maintained at 35 °C using a thermostatically controlled column oven. The flow rate was set at 0.3 mL/min, and a sample of 5 µL was injected for the analyses. The electrospray ionization (ESI) source was operated in positive-ion mode, and multiple reaction monitoring (MRM) was employed. The precursor–product ion pairs, 1082.6 *m/z* → 344.3 *m/z*, originated from azalomycin F_4a,_ were used for the detection. The fragmentor voltages and the collision energy were, respectively, set at 150 V and 70 V. The quantification analyses were validated by specificity, linearity, precision, accuracy, and recovery experiments (See Figure S1, Tables S1–S4 in Supplementary Materials).

### 2.4. HPLC Analysis and Method Validation

The concentrations of azalomycin F in biological samples from liver homogenate metabolism, everted intestinal sac experiments, and plasma protein-binding analyses were determined using a validated high-performance liquid-chromatographic (HPLC) method. Briefly, the quantitative analyses of azalomycin F were performed using a Waters e2695 separation system, consisting of a model 2998 ultraviolet detector (Waters, MA, USA), and the detection wavelength was set at 240 nm. A SinoChrom ODS-BP (4.6 mm × 250 mm, 5.0 µm, Elite, Dalian, China) was used as the chromatographic column and its temperature was kept at 25 °C. Methanol and ultrapure water at a ratio of 78:22 (*v*/*v*) was used as the mobile phase, and the flow rate was set at 1 mL/min. This analysis method was also validated by specificity, linearity, precision, accuracy, and recovery experiments (See Figures S2 and S3, Tables S1–S4 in Supplementary Materials). 

### 2.5. Preparation of Plasma, Live-Homogenate and Intestinal Sac Fluid Samples

Cold methanol of 400 μL (500 μL in the UPLC-MS/MS analyses) was added to 100 µL plasma or liver homogenate sample in a clean tube. The mixture was shaken by a vortex mixer for 1 min, and then centrifuged at 12,000 rpm for 10 min at 4 °C. After centrifugation, the supernatants of plasma or liver homogenate were filtered with filter membranes, and then a 10-µL aliquot was injected into the HPLC system for subsequent analysis (5 µL for UPLC-MS/MS). 

Four times volume of cold methanol was added into the intestinal sac fluid sample to precipitate proteins. According to the same operation above, the mixture was shaken, and then centrifuged to obtain the supernatant. Next, 400 μL of supernatant was dried under a gentle stream of nitrogen gas at 40 °C. The resulted residue was resuspended with 200 µL methanol, and then centrifuged at 12,000 rpm for 10 min at 4 °C. Finally, the upper liquid was filtered with filter membranes, and then a 10 µL aliquot was injected into the HPLC-UV system for subsequent analysis. 

### 2.6. Plasma Pharmacokinetics

After fasting overnight while freely drinking water for 12 h, ten male rats were divided randomly into two groups. Blood samples were respectively collected at set time point from the rats. For one group, azalomycin F at a dose of 26.4 mg/kg was administered to each rat by gavage. For another group, azalomycin F at a dose of 2.2 mg/kg was administered to each rat by intravenous injection. Blood samples (each 200 µL) were respectively collected into heparinized tubes from the post-orbital venous plexus veins of each rat at 0 (pre-dose), 10, 20, 40, 60, 120, 180, 240, 360, 480, 720, and 1440 min (total of 12 time points) after intragastric administration, and at 0 (pre-dose), 0.5, 2, 5, 10, 20, 40, 60, 120, 180, 240, 360, 480, 720, and 1440 min (total of 15 time points) after intravenous injection. Next, the blood samples were centrifuged at 4000 rpm for 10 min, and all resulting plasma samples were stored at −20 °C for subsequent analysis.

### 2.7. Intestinal Sac Absorption Test In Vitro (Everted Intestinal Sac Method)

Three rats were sacrificed and their necks broken, and the required intestinal segments were removed by rapid laparotomy. For each rat, three intestinal segments with 14 cm length were successively cut with the interval of 10 cm, from the distance of 10 cm to the pylorus, and marked as intestinal segments I, II and III. Another intestinal segment, 14 cm in length and marked as intestinal segment IV, was cut from a distance of 5 cm upward to the ileocecal valve. Immediately, these intestinal segments were placed in cold Tyrode’s solution. After the surface fat was removed, the intestinal segment was rinsed with cold Tyrode’s solution until the effluent was limpid, and then gently turned over. Next, one end of the intestinal segment was ligated, and 2 mL of blank Tyrode’s solution was injected into the intestinal sac. After this, the other end of the intestinal sac was also ligated. Finally, four intestinal sacs from each rat were placed in a beaker containing 50 mL of Tyrode’s solution, in which the concentration of azalomycin F was 0.20 mg/mL, and the solution was then bathed at 37 °C for 4 h. Simultaneously, a 1-mL aliquot, for the HPLC analysis, was taken from outside of the intestinal sacs at 1 h. After incubation for 4 h, the liquid mixture inside of the intestinal sacs was collected and stored at −20 °C. All samples above were processed according to the method described in Section 2.5 for further HPLC-UV analyses. 

### 2.8. Liver Homogenate Metabolism

Twenty-five percent (*m*/*v*) liver homogenate was obtained by the homogenization of rat liver with Tris-HCl-KCl solution, and stored at −80 °C. The experimental grouping was shown in Table 1, and all groups were incubated at 37 °C. Aliquots of the incubation mixture were taken after each group were incubated for 0, 3, 6, 10, 20, 32, 48, and 72 h. The concentration of azalomycin F in each mixture was analyzed using the HPLC-UV method mentioned above.

### 2.9. Stability of Azalomycin F in Plasma and Whole Blood

Thirty microliters of azalomycin F stock solution (10 mg/mL) were added into 1.2 mL plasma (or whole blood) to obtain the reaction system at an azalomycin F concentration of 0.25 mg/mL. This system was gently mixed and then incubated at 37 °C for 6 h. Next, 100 μL of the mixture was successively taken from the system after incubation for 0, 5, 10, 20, 40, 60, 120, 180, 240, 300, and 360 min, and processed to obtain the test sample according to the procedure described in Section 2.5. Finally, the concentrations of azalomycin F in the samples were determined by HPLC, and the results were expressed as mean ± standard deviation ($\bar{x}\pm s$).

### 2.10. Plasma Protein Binding Assay

According to previous method [33], the binding ratio of azalomycin F to plasma protein was determined by equilibrium dialysis. Briefly, 0.5 mL of blank plasma samples were placed in a dialysis bag, and then the bag was placed into 20 mL of PBS buffer (pH 7.4). Next, azalomycin F was added to the PBS to achieve final concentrations of 0.025, 0.05, and 0.1 mg/mL. These samples, laid on a shaker with the rotation speed of 160 rpm, were incubated at 37 °C for 24 h. Aliquots of 100 μL were simultaneously sampled from inside and outside of the dialysis bag, and then transferred to a 2.0 mL EP tubes to which 400 μL of cold methanol was added. Finally, the mixture was shaken in a vortex mixer, and next filtered with a filter membrane to obtain the sample for the HPLC–UV analysis. The plasma protein binding rate (*F_b_*) was calculated according to Equation (1) as follows:

(1) $$F_{b}\left(\%\right)\;=\;\frac{\left(D_{t}-D_{f}\right)}{D_{t}}\;\times \;100$$

where *D_t_* is the concentration of azalomycin F inside of the dialysis bag and *D_f_* is the concentration of azalomycin F outside of the dialysis bag.

### 2.11. Pharmacokinetic Parameters and Statistical Analysis

With the software Drug and Statistical Version 2.0 (DAS 2.0) (the Mathematical Pharmacology Committee, Chinese Pharmacological Society, Beijing, China), the pharmacokinetic parameters of azalomycin F administrated by oral and intravenous administrations were calculated by the non-compartment model. The maximum concentration (*C*_max_) of azalomycin F in plasma and the time to reach *C*_max_ (*T*_max_) were directly obtained from the experimental data. The areas under the curve from time zero to the last quantifiable concentration (AUC_0~t_) and those from time zero to infinity (AUC_0~∞_) were calculated using trapezoidal summation, respectively. Analyses of the experimental data were performed by one-way analysis of variance (ANOVA) using the software Data Processing System (DPS, College of Agriculture and Biotechnology, Zhejiang University, Hangzhou, China), and the results were expressed as mean ± SD (standard deviation).

## 3. Results

### 3.1. Method Validation

According to the method described in Section 2.3, quantitative analyses of azalomycin F in plasma were performed on an LC-MS/MS system, and the representative chromatographic profiles were presented in Figure S1 in Supplementary Materials. There was no obvious interfering peak derived from endogenous substances in the biological samples at the retention time of azalomycin F. The calibration curve showed that azalomycin F, at concentrations ranging from 15.6 to 500 ng/mL in the biological samples, presented good linearity with a correlation coefficient (r) of 0.9994. The intra- and inter-day precisions, expressed as relative standard deviation (RSD), were less than 6.67% and 11.23%, respectively. Accordingly, their accuracies were higher than 81.93% and 84.25%. The mean matrix effects were, respectively, 46.30%, 47.54% and 50.62% for azalomycin F at the concentrations of 15.6, 125, and 500 ng/mL. The mean extraction recovery of azalomycin F ranged from 89.02~104.74% (Tables S1–S3). 

Based on the methodological evaluation, the quantitative analyses for azalomycin F in rat plasma, liver homogenate, or in intestinal sac fluid were established and validated using HPLC-UV. Good separations of azalomycin F from contiguous peaks in various biological samples were achieved, and representative HPLC-UV profiles are shown in Figure S2 and Figure S3 in the Supplementary Materials. Simultaneously, good linearities between the concentrations and the chromatographic peak areas of azalomycin F are presented in Table S1, with all correlation coefficients (r) above 0.99. Furthermore, this analysis method also presented good intra- and inter-day precisions and accuracies (Table S2) and reliable recoveries (Table S3). Meanwhile, azalomycin F, under various storage conditions, was stable, as seen from the results in Table S4.

### 3.2. Pharmacokinetic Parameters

**Figure 1:**
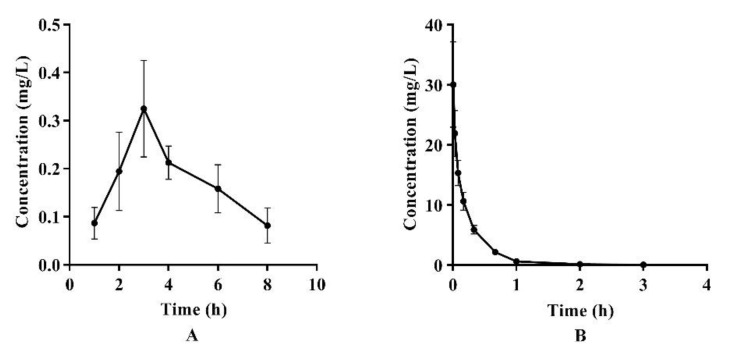
Mean plasma concentration–time curves of azalomycin F after (**A**) intragastric administration of 26.4 mg/kg and (**B**) intravenous administration of 2.2 mg/kg to rats.

The LC-MS/MS analysis was used to determine the concentrations of azalomycin F in plasma after a single intragastric administration of 26.4 mg/kg (*n* = 4) and a single intravenous administration of 2.2 mg/kg to the rats (*n* = 5). Their relevant pharmacokinetic parameters of azalomycin F were analyzed by the non-compartment model and these data are presented in Table 2. Simultaneously, the mean plasma concentration–time curves of azalomycin F, administrated by gavage and intravenous injection, are shown in Figure 1. The results show that azalomycin F ccould be absorbed after intragastric administration, while its absolute bioavailability (2.39%) was very low. Simultaneously, T_max_ and C_max_ were, respectively, 3 h and 0.325 mg/L, after azalomycin F was administrated by gavage. For intravenous administration, the back-extrapolated C_0_ was calculated as 4.561 mg/L.

### 3.3. Intestinal Sac Absorption Test In Vitro

The absorption ratios of azalomycin F in different intestinal segments in 4 h are shown in Table 3. From Table 3, the total absorption ratio of azalomycin F in four intestinal segments was 0.91% (*n* = 3), and the differences among these four intestinal segments presented as not significant (*p* > 0.05). Although the intestinal sac absorption test, in vitro, incompletely reflected the true absorption under normal physiological conditions, the above results still suggest that azalomycin F is difficult to absorb in the gastrointestinal tract, which is consistent with the very low absolute bioavailability of azalomycin F by intragastric administration. 

### 3.4. Liver Homogenate Metabolism Experiment

**Figure 2:**
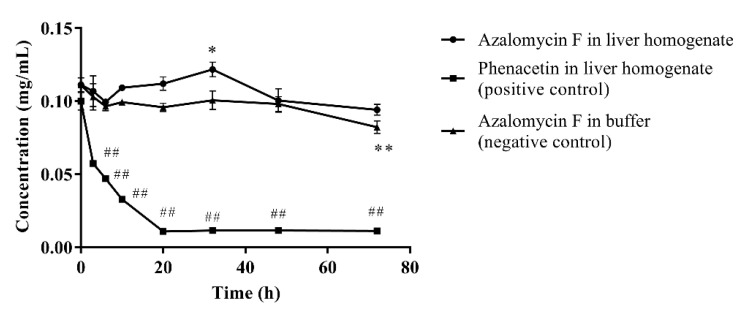
Metabolism of azalomycin F by liver homogenate (*n* = 3). ^##^ indicates that the residual concentration of phenacetine at a given time point in the positive control incubation system was significantly different from that at 0 h (*p* < 0.01); ** indicates that the residual concentration of azalomycin F at a given time point in the negative control incubation system was significantly different from that at 0 h (*p* < 0.01); * indicates that the residual concentration of azalomycin F at 32 h in the negative control incubation system was significantly different from that in liver homogenate (*p* < 0.05).

As show on Figure 2, ninety percent of phenacetin (positive control) was metabolized by liver homogenate (*p* < 0.01) after incubated at 37 °C for 72 h, and the chromatographic peak of the metabolite acetaminophen was visible in the HPLC profile. Compared to their individual initial concentrations, the concentration of azalomycin F in buffer remarkably decreased at 72 h (*p* < 0.01), but no significant decrease (*p* < 0.05) was observed in the liver homogenate. This suggests that not only is azalomycin F difficult for the liver to metabolize, but some non-enzymatic degradation to azalomycin F can be also inhibited. Considering the results of in Section 3.5 and Section 3.6, this is probably due azalomycin F’s binding to proteins in the liver homogenate, leading to the reduced degradation of microsomal enzymes of azalomycin F. 

### 3.5. The Stability of Azalomycin F in Plasma and Whole Blood

**Figure 3:**
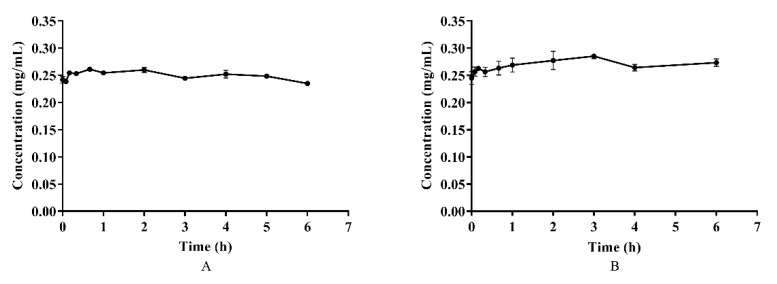
The stability of azalomycin F in plasma (**A**) and whole blood (**B**) (*n* = 3).

Azalomycin F, in plasma and whole blood, was incubated at 37 °C for 6 h, and the results are shown on Figure 3. Although a little fluctuation in the concentrations of azalomycin F in both plasma and whole blood was observed, as shown Figures 3A and B, there difference was insignificant (*p* > 0.05) for their individual concentrations during the incubation period, indicating that azalomycin F remains stable in both plasma and whole blood.

### 3.6. Plasma Protein Binding Assay

**Figure 4:**
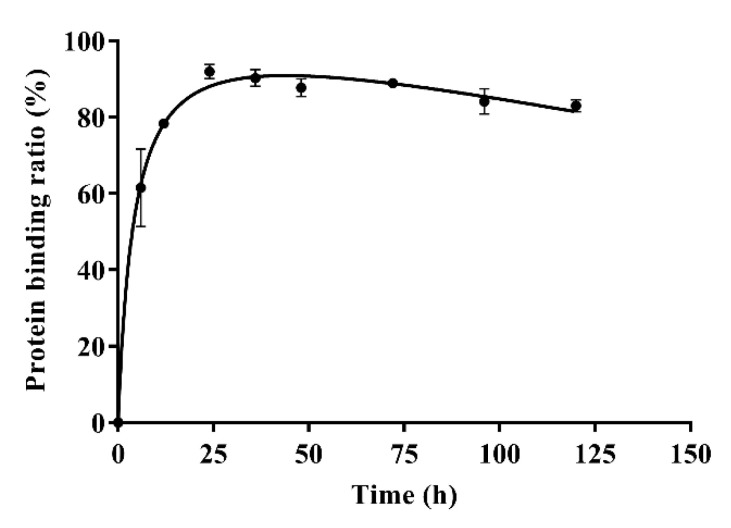
The plasma protein binding ratios of azalomycin F (0.05 mg/mL) at different time points (*n* = 3).

The results of in-vitro plasma protein binding are shown in Table 4. Form Table 4, the plasma protein binding ratios of azalomycin F were 96.88 ± 0.63%, 92.96 ± 0.76%, and 90.65 ± 0.92% when the concentrations of azalomycin F in PBS were 0.025, 0.05, and 0.1 mg/mL, respectively. Typical plasma protein binding ratios of azalomycin F (0.05 mg/mL) at different time points are shown in Figure 4. The results indicate that azalomycin F could bind to plasma proteins at low ratio in early time points and then reached a dynamic equilibrium at approximately 24 h. After 24 h, the plasma protein binding ratios slowly decreased. The dynamic change of protein binding may affect the pharmacokinetics of azalomycin F, especially at early time points. This may be the reason that the concentration of azalomycin F in blood decreased rapidly after it was administrated intravenously. Combined with the stability of azalomycin F in plasma, whole blood, and liver homogenate, we deduce that azalomycin F can rapidly distribute into the tissues or/and intracellular liquid from the blood of rats.

## 4. Discussion

Using UPLC-MS/MS technology, a rapid, specific and sensitive analysis method was developed for the quantitative determination of azalomycin F in rat plasma and used in the pharmacokinetics experiment. The HPLC analysis for the quantitative determination of azalomycin F in liver homogenate, intestinal sac fluid samples, and plasma protein binding of rats, in vitro, was also established. The results showed that the UPLC-MS/MS method was validated, using a dynamic calibration range between 15.6 and 500 ng/mL and a short run time of 4 min. For the HPLC-UV methods, the peak area and the concentration of azalomycin F had good linear relationships when the concentration of azalomycin F was 3.1~100.0 μg/mL over a run time of 20 min. Thereby, these analytical methods were validated for the quantitative determination of azalomycin F in the biosamples.

Based on these analytical methods, the pharmacokinetic characteristics and bioavailabilities of azalomycin F were investigated after it was administrated by gavage (26.4 mg/kg) and intravenous injection (2.2 mg/kg), respectively. As observable in Figure 1, the variance in the concentration of azalomycin F in blood samples collected from each rat, at the same time point was a little too great, so the non-compartment model was used for their pharmacokinetic analyses. The results showed that azalomycin F can be absorbed after administrated by gavage, and T_max_ and C_max_ were 3 h and 0.325 mg/L, respectively. However, its oral absolute bioavailability was very low (less than 5.0%). This indicates that azalomycin F is suitable for injection administration when used for systemic diseases, while local administration can be used for the treatment of diseases of the gastrointestinal tract.

Generally, bioavailability is directly related to membrane permeability, and may be affected by the presystemic metabolism derived from the gastrointestinal tract, and by enterohepatic circulation and gastric emptying [34,35]. The data in Figure 2 and Figure 3 indicate that azalomycin F has good stability in the liver homogenate, plasma, and whole blood. Simultaneously, HPLC analyses of the feces and urine (collected within 6–48 h) of the rats, after intravenous and oral administrations, indicated that azalomycin F can be excreted from the bile and detected in the feces, at 20.92% (orally) and 34.20% (intravenously) of the total administration amount (Table S5 and Table S6), while no prototype drug of azalomycin F was detected in the urine under both intravenous and oral administrations. Considering that the absorption ratio of intestinal sac for azalomycin F was very low (about 0.91%) (Table 3), the above suggests that the low oral absolute bioavailability of azalomycin F is due to the combined effects of the low absorption efficiency of azalomycin F in the intestinal tract, biliary excretion before systemic absorption into blood, and the degradation from both intestinal mucosa, during its absorption, and gut microorganisms, before fecal excretion. This may be also the reason that the acute toxicity of azalomycin F by gavage is much lower than that by intravenous administration [3]. Except for the degradation before the absorption, low absorption, and biliary excretion before systemic circulation, whether other presystemic metabolisms lead to the low oral absolute bioavailability of azalomycin F needs further research. Moreover, that the excretion amount from the feces for oral administration was about 12% higher than that for intravenous administration indicates that the possible degradation of azalomycin F happened in the stomach of rats.

Furthermore, it is unknown whether azalomycin F was metabolized by other tissues or organs, as only 34.20% of the dose for intravenous administration was excreted from feces. As the metabolites of azalomycin F were not assessed in this research, it remains unknown whether the feces and urine contained the metabolites of azalomycin F, although no proto-type drug of azalomycin F was detected in urine. In addition, it is worth noting that the degradation from intestinal mucosa and gut microorganisms may happen during azalomycin F’s movement from the common bile duct to the anus, as this would reduce the proportion of azalomycin F from biliary excretion as detected in the feces.

From Table 3, the intestinal sac absorption test, in vitro, indicated that it is difficult for azalomycin F to be absorbed. Simultaneously, the parameter t_1/2z_ (3.33 h) close to T_max_ (3 h) showed that azalomycin F absorption is slow after administrated by gavage, and which was also confirmed by the smaller absorption-rate constant Ka (0.168 h^−1^) (Table 2). These together indicate that azalomycin F can be absorbed by the intestinal tract at low degree and at a relatively slow rate. Another intestinal sac in-vitro absorption test indicated that azalomycin F can be absorbed at various intestinal segments without obvious difference, and which may be partly responsible for the greater value of the parameter T_max_.

Observed from Figure 1B, the mean plasma concentration rapidly dropped within 10 min after intravenous administration. Simultaneously, the back-extrapolated C_0_ (4.561 mg/L) was greatly lower than the ratio value of the dose to the volume of whole blood (about 34.375 mg/L). These above indicated that azalomycin F can be rapidly distributed into the tissues and/or intracellular liquid from the blood of rats, in vivo, after intravenous administration. Additionally, the low protein binding ratio in early time (Figure 4) can also abalienate enough time for azalomycin F to distribute to the tissues or organs.

From Table 2, two close CL values (0.341 and 0.412, respectively, for intravenous injection and gavage) predicted similar elimination for these two administrations: this is also supported by the similar slopes of their tail point-regression lines (0.181 for oral administration, and 0.190 for intravenous administration). However, other elimination routes remain unclear, except that the prototype drug can be excreted from the bile and feces, and that no prototype drug was detected in urine. As azalomycin F is stable in plasma, whole blood, and liver homogenate, where and how it is metabolized need further study, and, in vivo, its metabolites in rats should be also identified.

To enhance the solubility of azalomycin F, a surfactant of 0.5% CMC-Na was used for the dose formulation of intragastric administration, and this possibly delayed or reduced the absorption of azalomycin F, as it is viscous and can probably adsorb azalomycin F by electrostatic interaction. Simultaneously, a surfactant of 2% Tween was used for the dose formulation of the intravenous administration possibly delayed the distribution of azalomycin F from blood to tissues. Moreover, as the sensitivity of HPLC-UV used for the analyses of azalomycin F is lower than that of UPLC-MS/MS, a small amount of azalomycin F may not have been detected in the urine and feces. This would have reduced the total amount of azalomycin F excreted in the feces, and may have led to the absent detection of the prototype drug in the urine. However, it is nonetheless certain that biliary excretion is the main pathway of azalomycin F’s elimination in the form of prototype drug.

For ethical considerations, diethyl ether was administrated during the periods of blood sampling. According to previous publications [36,37], it is no obvious influence of diethyl ether on the rates of distribution and redistribution of drugs. However, this anesthesia can obviously inhibit the clearance of the drugs because it can interfere the metabolism and oxidation of the drugs in the liver. Therefore, diethyl ether, when used as an anesthetic in the pharmacokinetic research of drugs, is suggested for use with drugs with longer half-lives rather than those with short elimination half-lives [36]. Although azalomycin F presents short elimination half-lives (Table 2), the inhibition by diethyl ether of the clearance of drugs may be not suitable for studying azalomycin F, as it is stabile in the liver homogenate. However, the above suggest that there is little influence of diethyl ether in the distribution and elimination of azalomycin F.

As azalomycin F contains a side chain guanidine with a pKa value of 13 to 14 [38], it is completely protonated in the physiological environment and can maintain positive electricity in a large pH range [39,40]. Simultaneously, Figure 4 indicates that azalomycin F can bind to plasma proteins. Thereby, it was speculated that azalomycin F can also bind to α_1_-acidic glycoprotein in the liver homogenate and plasma through hydrogen bonding or electrostatic interaction, which may be responsible for the stability of azalomycin F in plasma, whole blood, and liver homogenate. 

## 5. Conclusions

The pharmacokinetics of azalomycin F were first investigated, and the plasma concentration time courses and pharmacokinetic parameters thereof, in rats, were obtained after azalomycin F was administrated by gavage (26.4 mg/kg) and intravenous injection (2.2 mg/kg). From this research, the following conclusions can be drawn:

(a) A rapid, specific and sensitive analysis method was developed using UPLC-MS/MS technology for the quantitative determination of azalomycin F in rat plasma, and the HPLC analysis for the quantitative determination of azalomycin F in the liver homogenate, intestinal sac fluid samples, and plasma protein binding of rats, in vitro, was also established.
(b) After administrated by gavage, azalomycin F can be absorbed by intestinal tract at low degree and relatively slow rate, and its absolute bioavailability is very low. This indicated that azalomycin F is suitable for intravenous administration when used for systemic diseases, while oral administration can be used for the treatment on the diseases of gastrointestinal tract.
(c) The low oral absolute bioavailability of azalomycin F is likely due to the combined effects of its low absorption efficiency in the intestinal tract, the bile excretion before the absorption into the systemic blood, and the degradation from both intestinal mucosa, during its absorption, and gut microorganisms, before fecal excretion. This may be also the reason that the acute toxicity of azalomycin F by gavage was much lower than that by intravenous administration.
(d) After administrated by intravenous injection or absorbed from the intestinal tract, azalomycin F can be rapidly distributed into the tissues and/or intracellular fluid from the blood of rats.
(e) Azalomycin F presents plasma protein binding ratios of more than 90% and is stable in plasma, whole blood, and liver homogenate. The last is likely due to the binding between azalomycin F and α_1_-acidic glycoprotein in the liver homogenate and plasma.
(f) Biliary excretion is the major pathway of eliminating azalomycin F in the form of a prototype drug, and no prototype drug was detected in the urine, while other elimination routes remain unclear. Therefore, the metabolic sites and identifications of azalomycin F metabolites should be further explored for.

## Figures and Tables

**Table 1:** The grouping of liver homogenate experiment (*n* = 3).

Group Name	Detail ^a^
Blank	10 μL methanol + 1 mL liver homogenate
Negative control	10 μL 10 mg/mL Azalomycin F solution + 1 mL Tris-HCl-KCl solution
Positive control	10 μL 10 mg/mL phenacetin solution + 1 mL liver homogenate
Experimental group	10 μL 10 mg/mL Azalomycin F solution + 1 mL liver homogenate

^a^: Both azalomyin F and Finacetine solutions were respectively prepared using methanol.

**Table 2:** Main pharmacokinetic parameters of azalomycin F_4a_ after intragastric administration of 26.4 mg/kg and intravenous administration of 2.2 mg/kg to rats (mean ± SD) ^a^.

Parameters	Units	26.4 mg/kg (i.g.)	2.2 mg/kg (i.v.)
AUC_(0-t)_	mg/L∙h	1.380 ± 0.544	6.474 ± 0.886
AUC_(0-∞)_	mg/L∙h	1.873 ± 1.007	6.541 ± 0.851
*V*_Z_	L/kg	1.691 ± 0.359	0.434 ± 0.679
*t*_1/2_	h	3.329 ± 1.477	0.945 ± 1.516
*CL*	L/h/kg	0.412 ± 0.207	0.341 ± 0.050
MRT_(0-∞)_	h	6.458 ± 2.333	0.490 ± 0.435
*C*_max_	mg/L	0.325 ± 0.200	-
*T*_max_	h	3	-
*C*_0_ ^b^	mg/L	-	4.561
K_a_ ^c^	1/h	0.168	-
F	%	2.386 ± 1.283	-

^a^: With the software Drug and Statistical Version 2.0, the pharmacokinetic parameters of azalomycin F, administrated by gavage (*n* = 4) and intravenous injection (*n* = 5), were calculated by the non-compartmental model. ^b^: The C_0_ value was calculated from the back-extrapolation of elimination phase. ^c^: The K_a_ value was calculated as the reciprocal of the MRT_(0-∞)_ difference between gavage and intravenous injection.

**Table 3:** Absorption ratio of azalomycin F in four intestinal segments (*n* = 3).

	Segment I	Segment II	Segment III	Segment IV	Total
Absorptive amount (mg)	0.021 ± 0.009	0.022 ± 0.015	0.025 ± 0.022	0.022 ± 0.011	0.090 ± 0.048
Absorption ratio (%)	0.21 ± 0.09	0.23 ± 0.15	0.26 ± 0.24	0.22 ± 0.11	0.91 ± 0.51

**Table 4:** The plasma protein binding ratios of azalomycin F at 24 h (*n* = 3).

Added Concentration (mg/mL)	Concentration in Plasma (mg/mL)	Concentration in Buffer (mg/mL)	Protein-Binding Ratio (%)
0.025	0.495 ± 0.055	0.015 ± 0.001	96.88 ± 0.63
0.05	0.675 ± 0.043	0.047 ± 0.004	92.96 ± 0.76
0.1	1.062 ± 0.115	0.098 ± 0.008	90.65 ± 0.92

## Data Availability

Not applicable.

## Supplementary Materials

The following are available online, Figure S1: Representative multiple reaction monitoring (MRM) chromatograms in rat plasma of blank samples (I), blank samples containing LLOQ (15.6 ng/mL) of azalomycin F (II), the obtained samples at 40 min after a single oral administration of azalomycin F (2.2 mg/kg) (III). Figure S2: Representative HPLC-UV chromatograms in rat plasma (A), whole blood (B) of blank samples (I), blank samples containing LLOQ of azalomycin F (II), the obtained samples in plasma and whole blood stability test (III). (A) plasma (I: blank, II: LLOQ, III: the obtained sample at 5 h after incubated in plasma); (B) whole blood (I: blank, II: LLOQ, III: the obtained sample at 5 h after incubated in whole blood). Figure S3: Representative HPLC-UV chromatograms in rat liver homogenate (A), intestinal sac fluid samples (B) of blank samples (I), blank samples containing LLOQ of azalomycin F (II), the obtained samples in liver homogenate metabolism experiment and intestinal sac absorption test (III). (A) liver homogenate (I: blank, II: LLOQ, III: the obtained sample at 17 h after incubated in liver homogenate); (B) intestinal sac fluid samples (I: blank, II: LLOQ, III: the obtained sample at 4 h after in vitro intestinal absorption). Table S1: Standard curve, correlation coefficient and the limit of quantification of azalomycin F (*n* = 3). Table S2: The precision and accuracy of the intra- and inter-day analyses (*n* = 5). Table S3: The extraction recovery and matrix effect of the analysis methods (*n* = 5). Table S4: Stability of azalomycin F under various storage conditions (*n* = 5). Table S5: Mean feces accumulative excretion amount of azalomycin F after single oral administration (26.4 mg/kg) evaluated by the HPLC-UV method in rats (*n* = 3). Table S6: Mean feces accumulative excretion amount of azalomycin F after single intravenous administration (2.2 mg/kg) evaluated by the HPLC-UV method in rats (*n* = 3).

## Sample Availability

Samples of the compound azalomycin F are available from the authors.
