# Supplementary material for: Pharmacokinetics of Azalomycin F, a Natural Macrolide Produced by Streptomycete Strains, in Rats
Source: Molecules. 2021 Oct 26;26(21):6464. doi: 10.3390/molecules26216464 (PMC8588360; doi:10.3390/molecules26216464)
Supplement: Supplementary file 1 [file molecules-26-06464-s001.zip › molecules-1428389-supplementary.pdf]

molecules

MDPI

Supplementary Files

Pharmacokinetics of Azalomycin F, a Natural Macrolide Produced by Streptomycete Strains, in Rats

Su He [1], Wenjia Zhao [1], Peibo Li [2], Wenqing Tu [3], Kui Hong [4], Duoduo Zhang [1], Tongke Zhang [1], Ganjun Yuan [1,*]

[1] Biotechnological Engineering Center for Pharmaceutical Research and Development, Jiangxi Agricultural University, Nanchang 330045, China; suhepeilin@gmail.com (S.H.); 18770910458@sohu.com (W.Z.); blossom_zhang@sohu.com (D.Z.); ztk2628925331@sohu.com (T.Z.)

[2] Guangdong Engineering and Technology Research Center for Quality and Efficacy Re-evaluation of Post-marketed TCM, State Key Laboratory of Biocontrol and Guangdong Provincial Key Laboratory of Plant Resources, School of Life Sciences, Sun Yat-sen University, Guangzhou 510275, China; li-peibo@mail.sysu.edu.cn

[3] Research Institute of Poyang Lake, Jiangxi Academy of Sciences, Nanchang, 330012, China; tuwen-qing@jxas.ac.cn

[4] Key Laboratory of Combinatorial Biosynthesis and Drug Discovery, Ministry of Education, School of Pharmaceutical Sciences, Wuhan University, Wuhan 430071, China; kuihong31@whu.edu.cn

* Correspondence: gyuan@jxau.edu.cn (G.Y.); Tel.: +86-0791-83813459

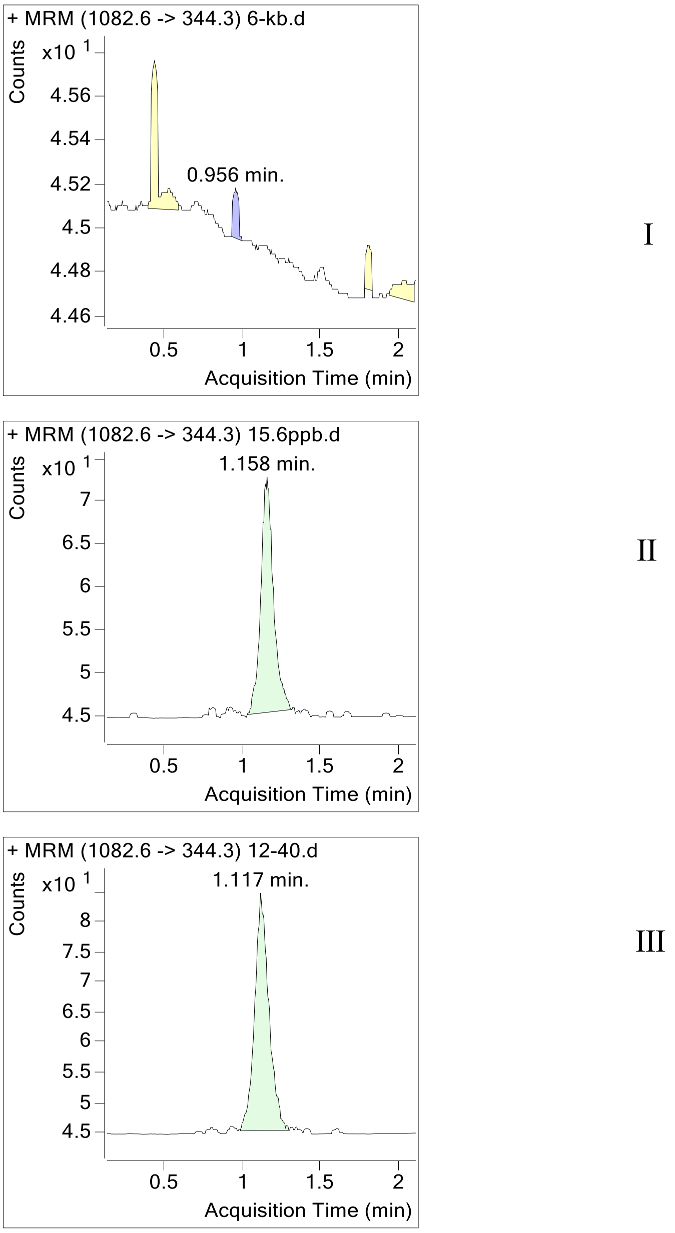

Figures S1. Representative multiple reaction monitoring (MRM) chromatograms in rat plasma of blank samples (I), blank samples containing LLOQ (15.6 ng/mL) of azalomycin F (II), the obtained samples at 40 min after a single oral administration of azalomycin F (2.0 mg/kg) (III).

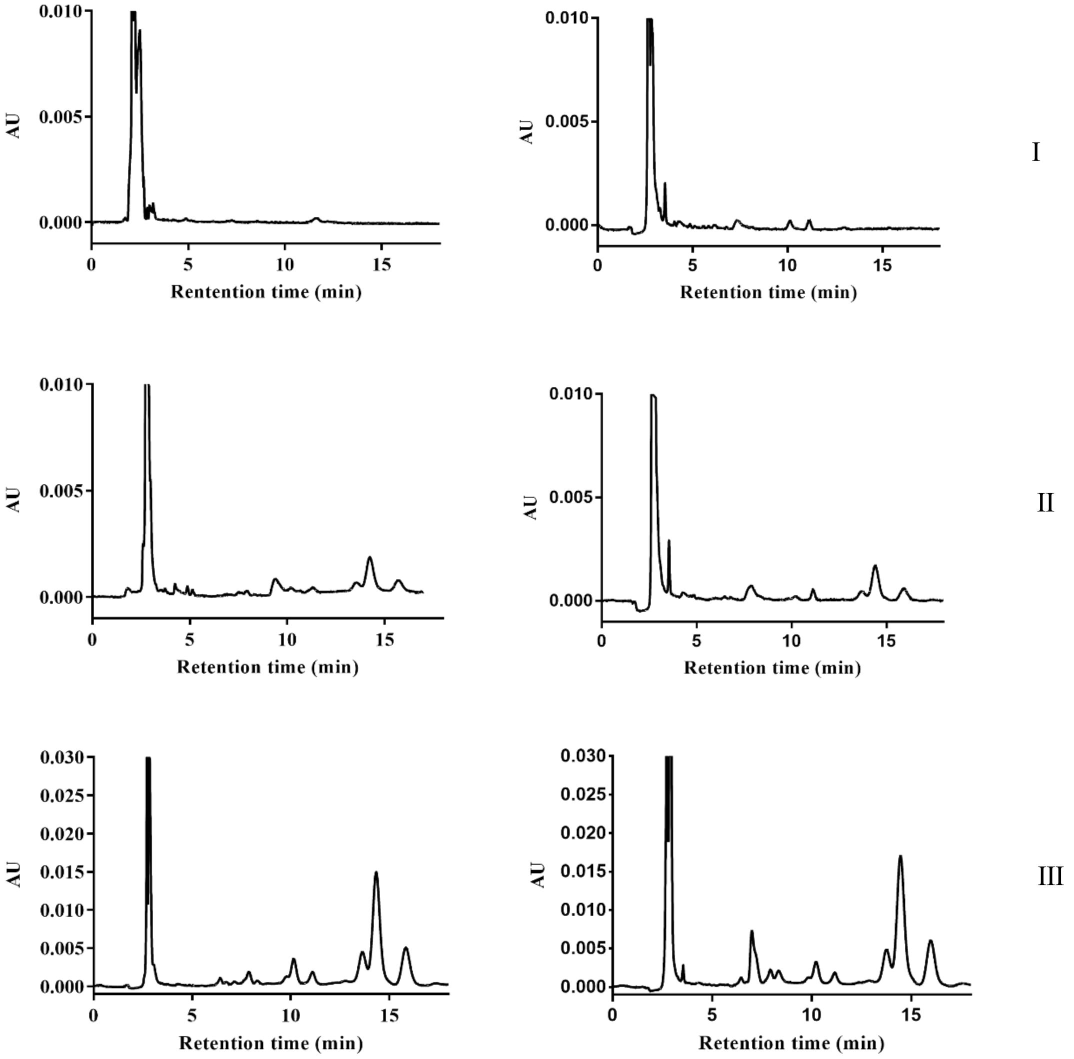

A B

Figures S2. Representative HPLC-UV chromatograms in rat plasma (A), whole blood (B) of blank samples (I), blank samples containing LLOQ of azalomycin F (II), the obtained samples in plasma and whole blood stability test (III). (A) plasma (I: blank, II: LLOQ, III: the obtained sample at 5 h after incubated in plasma); (B) whole blood (I: blank, II: LLOQ, III: the obtained sample at 5 h after incubated in whole blood).

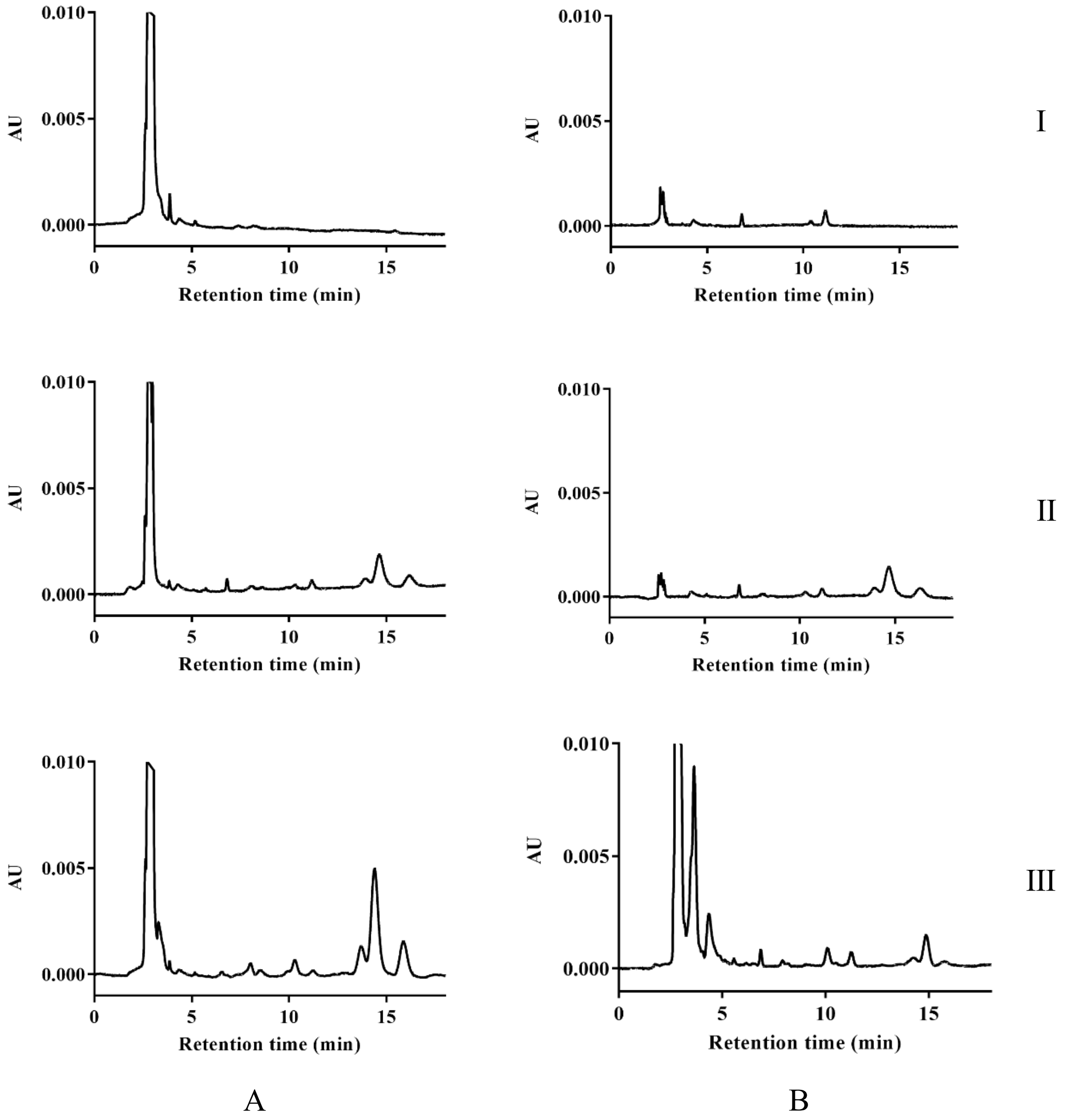

Figures S3. Representative HPLC-UV chromatograms in rat liver homogenate (A), intestinal sac fluid samples (B) of blank samples (I), blank samples containing LLOQ of azalomycin F (II), the obtained samples in liver homogenate metabolism experiment and intestinal sac absorption test (III). (A) liver homogenate (I: blank, II: LLOQ, III: the obtained sample at 17 h after incubated in liver homogenate); (B) intestinal sac fluid samples (I: blank, II: LLOQ, III: the obtained sample at 4 h after in vitro intestinal absorption).

Table S1. Standard curve, correlation coefficient and the limit of quantification of azalomycin F (n=3)

Biological Samples	Standard curve	Correlation coefficient (r)	The limit of quantifications（LOQs）
Plasma	$y = 1*10^7x + 60260$	0.9962	3.12 μg/mL
Whole blood	$y = 2*10^7x + 39610$	0.9989	3.12 μg/mL
Liver homogenate	$y = 1*10^7x + 67037$	0.9991	3.12 μg/mL
Intestinal sac fluid	$y = 1*10^7x + 50772$	0.9976	3.12 μg/mL
Plasma*	$y = 5.8412*x + 96.537$	0.9994	15.6 ng/mL

*: The sample of plasma was evaluated by ultra-high performance liquid chromatography tandem mass spectrometry (UPLC–MS/MS) method.

Table S2. The precision and accuracy of the intra- and inter-day of the analysis method (n=5)

		Intra- Day			Inter- Day		
Biological Samples	Nominal Concentration (μg/mL or ng/mL)	Measured concentration (μg/mL or ng/mL)	Accuracy (%)	Precision (RSD, %)	Measured concentration (μg/mL or ng/mL)	Accuracy (%)	Precision (RSD, %)
Plasma	3.12	2.88	92.31	9.71	2.81	90.06	7.83
	12.5	12.25	97.97	4.32	11.62	93.00	6.97
	100	101.82	101.82	1.88	94.88	94.88	5.74
Blood	3.12	3.27	104.90	9.44	3.26	104.56	9.77
	12.5	12.77	102.13	4.18	13.09	104.70	4.61
	100	94.54	94.54	7.02	99.09	99.09	6.40
Liver homogenate	3.12	3.11	99.56	5.03	3.11	99.74	5.15
	12.5	12.80	102.38	3.37	12.55	100.40	3.05
	100	106.83	106.83	2.15	103.20	103.20	4.73
Intestinal sac fluid	3.12	3.04	97.58	2.49	2.97	95.12	3.38
	12.5	12.64	101.12	6.82	12.62	100.94	3.92
	100	95.98	95.98	2.41	95.72	95.72	2.32
Plasma*	15.6	12.78	81.93	4.34	13.16	84.25	8.32
	125	107.05	85.64	5.14	107.17	85.73	10.13
	500	504.06	100.81	6.67	504.03	100.81	11.23

*: The sample of plasma was evaluated by ultra-high performance liquid chromatography tandem mass spectrometry (UPLC–MS/MS) method.

Table S3. The extraction recovery and matrix effect of the analysis methods (*n*=5)

Biological Samples	Nominal Concentration (μg/mLor ng/mL)	Matrix Effect (%)	Extraction Recovery (%)
Plasma	3.12	107.58	97.08
	12.5	101.29	105.86
	100	103.81	106.57
Blood	3.12	108.54	96.64
	12.5	105.27	97.02
	100	108.00	87.54
Liver homogenate	3.12	100.27	100.72
	12.5	101.56	100.01
	100	98.92	98.11
Intestinal sac fluid	3.12	95.78	101.88
	12.5	93.38	108.29
	100	89.46	107.28
Plasma*	15.6	46.30	104.74
	125	47.54	95.46
	500	50.62	89.02

*: The sample of plasma was evaluated by ultra-high performance liquid chromatography tandem mass spectrometry (UPLC–MS/MS) method.

Table S4. Stability of azalomycin F under various storage conditions (HPLC-UV method, *n*=5)

Biological Samples	Concentration (μg/mL)	room temperature for 24 h		Post- preparative stability (at 4°C for 48 h)		Freeze- thawing three cycles (at −20°C)		Freezing storage (at - 20°C for 3.5 months)	
		Accuracy (%)	RSD (%)	Accuracy (%)	RSD (%)	Accuracy (%)	RSD (%)	Accuracy (%)	RSD (%)
Plasma	3.12	101.03	8.53	99.01	8.28	95.99	5.14	104.11	13.91
	12.5	105.80	4.07	97.69	8.69	97.80	4.22	108.38	7.32
	100	100.84	2.63	91.33	2.90	89.73	1.51	103.68	2.22
Blood	3.12	94.78	9.69	100.23	9.78	83.58	7.04	105.36	3.47
	12.5	92.45	4.52	106.71	5.11	87.56	3.35	90.46	3.89
	100	95.67	6.29	94.73	7.26	87.64	0.69	100.75	1.87
Liver homogenate	3.12	101.00	3.56	98.99	5.03	98.51	7.34	90.12	3.16
	12.5	101.57	3.31	100.98	3.37	98.66	2.59	95.08	2.52
	100	97.04	2.32	100.40	2.15	99.86	2.51	100.89	1.60
Intestinal sac fluid	3.12	95.37	3.75	94.29	2.92	98.17	1.76	91.57	6.07
	12.5	93.46	6.94	100.39	0.88	99.06	2.26	91.81	2.33
	100	96.12	3.47	99.77	2.42	99.41	2.65	94.41	5.10

Table S5. Mean feces accumulative excretion amount of azalomycin F after single **oral administration** (26.4 mg/kg) evaluated by the HPLC-UV method in rats (n=3, mean ± SD) [a]

**Time Point (h)**	**6-12**	**12-24**	**24-48**	**Accumulative Excretion**
Excretion Amount (mg)	0.516 ± 0.430	0.363 ± 0.174	0.324 ± 0.223	1.381 ± 0.830
Percentage of dose (%)	7.82 ± 6.52	5.5 ± 2.64	4.91 ± 3.38	20.92 ± 12.58

[a]: Feces at each time period was collected to a volumetric flask of 100 mL, and about 90 to 95 mL methanol was added to. Then, the mixture was sonicated for 15 min, and a small amount of methanol was added to tick mark. Finally, the mixture was mixed well, and the supernatant was filtered through a filter membrane (0.22 μm) to obtain the test sample for HPLC-UV analyses according to the method as "2.4. HPLC Analysis and Method Validation.

Table S6. Mean feces accumulative excretion amount of azalomycin F after single **intravenous administration** (2.0 mg/kg) evaluated by the HPLC-UV method in rats (n=3, mean ± SD) [a]

**Time Point (h)**	**6-12**	**12-24**	**24-48**	**Accumulative Excretion**
Excretion Amount (mg)	0.082 ± 0.023	0.054 ± 0.024	0.035 ± 0.030	0.171 ± 0.077
Percentage of dose (%)	16.40 ± 4.60	10.80 ± 4.80	7.00 ± 6.00	34.20 ± 15.40

[a]: Feces at each time period was collected to a volumetric flask of 100 mL, and about 90 to 95 mL methanol was added to. Then, the mixture was sonicated for 15 min, and a small amount of methanol was added to tick mark. Finally, the mixture was mixed well, and the supernatant was filtered through a filter membrane (0.22 μm) to obtain the test sample for HPLC-UV analyses according to the method as "2.4. HPLC Analysis and Method Validation.
